# Cervical Cancer Awareness and Attitude Towards Cervical Cancer Screening and Human Papillomavirus Vaccines Among Urban Women of Karachi, Pakistan

**DOI:** 10.7759/cureus.42970

**Published:** 2023-08-04

**Authors:** Saira Shahnaz, Eduardo Fricovsky, Ramesha Anwar, Mudassar Iqbal Arain

**Affiliations:** 1 Pharmacy, Nazeer Hussain University, Karachi, PAK; 2 Clinical Pharmacy, Skaggs School of Pharmacy and Pharmaceutical Sciences, La Jolla, USA; 3 Pharmacy, University of Karachi, Karachi, PAK; 4 Pharmacy, University of SIndh, Jamshoro, PAK; 5 Pharmacy, Skaggs School of Pharmacy and Pharmaceutical Sciences, La Jolla, USA

**Keywords:** pakistan, karachi, hpv vaccination, pap-smear, cervical cancer

## Abstract

Cervical cancer is the second most common cancer among women under 50 years of age in Pakistan. The current study was designed to assess the level of awareness through educational outreach presentations about cervical cancer, Papanicolaou (Pap) smear test, and human papillomavirus (HPV) vaccination in Karachi, Pakistan. Women from different urban hospitals were enrolled. Participants participated in a 45-minute presentation on cervical cancer awareness led by student pharmacists from Nazeer Hussain University, Karachi. A pre-and post-test was administered to assess the impact of the intervention. Descriptive statistics were used to summarize the findings, and a t-test was used for matched comparison, and a p-value <0.05 for statistical significance. A total of 150 women participated in the study. The study found that Pakistani women living in urban settings were less knowledgeable about the causes of cervical cancer and prevention. After the presentation, we observed a 45% increase in knowledge, and 31% of participants said they would obtain a Pap smear test in the next six months. Supervised pharmacy student-led presentations on cervical cancer educational awareness that significantly impacted women participants. Pharmacists can play a key role in reducing cervical cancer deaths through increased awareness, education, prevention, and immunization.

## Introduction

According to the WHO, cervical cancer is the 4th most common cancer and third most common cause of death affecting women globally. In 2020, over 600,000 women were diagnosed, and 342,000 women died from cervical cancer worldwide. 90% of all new cases and deaths occurred in low- and middle-income countries [1]. Pakistan ranks as the 5th most populous country in the world. It is in South Asia and is classified as a low-income and developing country [2-3]. Pakistan's health care system consists of both public and private sectors, but 70% of the population receives care from the private sector, and 85% is uninsured [4]. According to the WHO, in 2021, over 5000 Pakistani women were diagnosed with cervical cancer, and 3006 deaths were reported. These numbers are believed to be low because there is no current national program for cervical cancer screening.

Further, only 1 in 10 Pakistani have been screened for cervical cancer in the past five years. Overall, in 2019 less than 1% of women in Pakistan reported cervical cancer-screening tests [5]. Worldwide, more than 70% of cervical cancers are caused by two strains of human papillomavirus (HPV) 16 and 18. These strains are also called high-risk strains of HPV [6-7]. The prevalence of high-risk strains of HPV was common in women in developing countries. In Pakistan, the percentage was reported to be up to 90% [8-9]. To combat cervical cancer and/or new HPV infections, WHO has recommended that HPV vaccines should be administered to young children before first sexual contact. The HPV vaccines were first approved in 2006 in the United States of America and Europe, respectively, and licensed in more than 100 countries, including Pakistan [10]. Pakistan has a national Extended Program Immunization (EPI) registry to track the administration of vaccines, however, it does not track HPV vaccines. Several smaller studies have found that HPV vaccination rates among Pakistani women were low [1]. Pakistan's level of cervical cancer cases and mortality is growing. Educational, preventive, and screening measures must be implemented nationwide [11]. Currently, there are few awareness and preventive efforts in parts of the country. Raising awareness about the benefits of screening and HPV vaccines has motivated women to obtain a Pap-smear test and/or HPV vaccinations [12].

We designed the study to assess the effect of community-based student pharmacist-run educational outreach on cervical cancer awareness and attitude towards Pap smears and HPV vaccines among urban low-income women of Karachi, Pakistan. To our knowledge, this is the first of its kind effort in the country and has been previously shown in other countries to have a significant and positive impact on women's knowledge and attitudes towards Pap-smear testing and HPV vaccines.

## Materials and methods

Study design

A descriptive, cross-sectional, and interventional study was conducted to assess the effect of an educational outreach program by student pharmacists under a qualified pharmacist about awareness of cervical cancer and to analyze the current trend of attitude towards Pap smear tests and HPV vaccines among women of Karachi, Sindh, Pakistan.

Study subjects

Based on a random sampling technique, 150 women were enrolled from different hospitals in Karachi and invited to participate in a 45-minute presentation on cervical cancer awareness, Pap smear tests, and HPV vaccination. The current study objective was to assess the level of knowledge about cervical cancer signs and symptoms, prevention, and early detection and outcomes. Our second objective was to measure attitudes toward obtaining a Pap smear test in the next 6 months. The outreach awareness presentation was held in the auditorium of NHU, Karachi, in August 2022. Further, patients over 18 years without any disease discrimination were included in the study.

Study instrument

Pre- and post-test questionnaires were conducted to analyze the effects of the intervention. In addition, demographic information was collected, including age, gender, weight, education level, health insurance status, marital status, co-morbidities, and smoking status. Adopting this study design is more practical due to our limited budget, workforce, time, and space constraint to conduct the study. Moreover, the pre-and post-test study design has been shown to be effective in studying educational interventions. Participants were handed consent to read, sign, and date. Participants were then asked to fill out a pre-questionnaire before the presentation's start and at the end. The questionnaires were 10 minutes in length each. The presentation consists of basic information about cervical cancer, signs and symptoms, screening, detection, risks, prevention (HPV vaccines), and information about Pap smear tests. The presentation was done in the local language Urdu using layperson's terms and avoiding medical jargon. Questionnaires were distributed in English, Urdu, or Sindhi based on participants' language preferences.

Ethics statement

The institutional review board of Nazeer Hussain University, Karachi, Pakistan, vide letter number (2022/321) approved the study. Written consent was obtained from the patients described in the study.

Statistical analysis

The study was described descriptively with the help of different software such as Microsoft Excel, SPSS Version 20, and JMP Version 16. A t-test statistical analysis was performed comparing pre- and post-questionnaire responses, and p<0.05 was considered statistically significant.

## Results

Demographic and clinical data description

We enrolled 150 women to participate in a 45-minute presentation about cervical cancer awareness. We conducted a pre- and post-questionnaire, 10 minutes in length each. The overall response rate was 89%. The average median age was 52 years old. 30% of the participants had not received a formal primary education, 28% reported some primary education (1st- 5th grade), and only 8% reported having attended college or university. 98% of participants reported being married and clinically had one or more co-morbid conditions such as hypertension and diabetes mellitus Type 2. Only 16% of participants reported having health insurance. The details of participants' demographics and clinical descriptions are described in Table 1.

**Table 1:** Demographic and clinical data of participants. The details of participants’ demographics and clinical description.

Variable		Response N (%)
No. Participants		150
Age, median, years		52
Age ranges, n (years), %	40-49	64 (43)
	50-59	59 (39)
	60-64	15 (10)
	> 65 years	12 (8)
Weight, median [IQR], kg		71 (56,77)
Education	Uneducated	45 (30)
	Primary (1-5th grade)	43 (29)
	Secondary (6-10th grade)	29 (19)
	College (11-12th grade)	21 (14)
	University (Bachelor/ Master)	12 (8)
Marital Status	Married	138 (92)
	Unmarried	5 (3)
	Divorced	7 (5)
Co-Morbid Conditions	Diabetes	59 (39)
	Hypertension	50 (33)
	Arthritis	2 (1)
	No any	39 (26)
Smoking Status	Yes	43 (29)
	No	107 (71)
Health Insurance	Yes	24 (16)
	No	126 (84)

Before the intervention, only 19% of participants reported reading or talking to someone about cervical cancer and 23% about Pap smear tests. 82% of participants stated that fear was the top reason why they did not plan to get a Pap-smear test; other factors included embarrassment (87%) and cost (77%). About 59% of women responded "that they did not believe in the test." After the presentation, all of the reasons for avoiding a Pap smear test significantly dropped in percentages, such as fear (66%) and embarrassment (51%), including the percentage of reposes saying, "did not believe in the test" (27%). One of the factors that did not change in responses was the lack of female doctors (Tables 2, 3).

**Table 2:** Questions regarding cervical cancer awareness?

Questions	Yes (n,%)	No (n,%)
Have you ever read, or has anyone talked to you about cervical cancer?	28 (19)	122 (81)
Have anyone talked to you, or have you read about the Pap smear test?	35 (23)	115 (77)

**Table 3:** Cervical cancer screening attitude. *p < 0.05, ** p < 0.01, and ***p <0.001

Variable	Pre-Seminar (N=150)		Post-Seminar (N=134)	
	Frequency	Percentage	Frequency	Percentage
It might be painful/uncomfortable.	65	43%	32	24%***
Fear	123	82%	88	66%**
Embarrassment	117	78%	68	51%***
Don’t have a female doctor.	39	26%	39	29%
Don’t believe in Pap smear.	88	59%	36	27%***
Too costly	115	77%	68	51%***
Don’t know where to go for it	57	38%	21	16%***
Insurance won’t pay for it.	22	15%	6	5%**
Don’t have time to go see a doctor	66	44%	33	25%***
Had Cervix removed (hysterectomy)	14	9%	14	10%

Cervical cancer knowledge

Knowledge assessment of participants about cervical cancer and prevention was low before the presentation. When asked, "Do women with cervical cancer show pain or discomfort?" 100% of participants responded that they "don't know." Similarly, when asked "if HPV vaccination is a method for preventing cervical cancer?" all of the participants responded, "don't know." When asked if "cervical cancer is treatable in the early stage?" 8% answered "Yes". After the presentation, knowledge about cervical cancer and prevention increased significantly. 35% of participants were aware of symptoms of cervical cancer, 57% answered "Yes" that HPV vaccines can be used to prevent cervical cancer, and 47% responded "Yes" that cervical cancer is treatable in the early stages (Table 4).

**Table 4:** Assessment of knowledge about cervical cancer and prevention. *p < 0.01; 45% overall increase in knowledge from baseline.

Questions	Pre-Seminar (N=150)			Post-Seminar (N=134)		
	Yes(n,%)	No,(n,%)	Don’t Know (n,%)	Yes (n,%)	No, (n,%)	Don’t Know(n,%)
Do women with cervical cancer show pain or discomfort?	0	0	150 (100)	47(35)*	31 (23)	56(42)
Human Papilloma Virus (HPV) vaccination is a method for preventing cervical cancer?	0	0	150 (100)	76 (57)*	37 (28)	21 (16)
Cervical cancer is treatable in early stage?	12 (8)	29 (19)	109 (81)	63 (47)*	36 (27)	35 (26)
Average	8%			53%		

Prior to the intervention, 54% of the participants stated "No" to any plan to have a Pap smear in the coming 6 months. 46% responded, "don't know." After the presentation, we saw a significant shift in the percentage, 31% of participants responding "Yes" and said they plan to have a Pap smear test in the next 6 months. However, despite the intervention, 38% responded, "No" and 30% "Don't know" (Table 5).

**Table 5:** Any plan to have a Pap smear test in the coming six months. *p < 0.01

Variable	Pre-Seminar (N=150)		Post-Seminar (N=134)	
	Frequency	Percentage	Frequency	Percentage
Yes	0	0	42	31.34%*
No	81	54	51	38.05%
Don’t know	69	46	41	30.60%

## Discussion

Cervical cancer is a preventable disease. HPV vaccination helps to prevent infection from HPV viruses that cause cervical cancer. Pap-smear testing screens for cervical cancer abnormal cells and, if detected early, can be successfully treated. Poor knowledge and lack of access to preventable care in low-income women in underdeveloped countries contribute to poor outcomes and deaths. Our study aimed to assess the knowledge and attitudes of cervical cancer, screening, and HPV vaccination and prevention before and after an educational intervention in urban communities of Karachi, Pakistan.

We found that most women who participated in the study lacked health insurance. The lack of health insurance is a major reason women in developing countries do not obtain Pap smear screening tests [13]. According to the Pakistan Institute of Development Economics (PIDE), only 15.7% of Pakistanis have health insurance coverage [14]. The number of participants in our study with health insurance closely reflected this statistic. Low education is another common factor contributing to low Pap smear tests among women in developing countries. Corral et al. [15] reported that those women in Ecuador who had primary or less primary education had more than twice the chance of developing cervical cancer as compared to those women who had secondary education.

Further, it was also discussed in another study that the impact of no education was that those women who had no education were more likely to develop cervical cancer, and the chances were six times as compared to the previous one [16]. Our study did not assess the level of cancer incidence among participants, but we did find a low education level among women (30% reporting no primary education and 29% some primary education), which puts them at higher risk for cervical cancer. Another cervical cancer risk factor is smoking. Vaccarella et al., in 2008, pooled an analysis of 13 HPV prevalence surveys in 11 countries worldwide, carried out between 1993 and 2005. They concluded that current tobacco smoking was associated with a significant, although moderate, increased risk of prevalent HPV infection [17]. Our study found that 29% of participants are current tobacco smokers, which increases their risk for HPV infections. Referring to tobacco cessation programs that offer tobacco cessation products could help reduce the risk of prevalent HPV infections in this sub-group [18].

We also assess participants' knowledge regarding awareness of cervical cancer and prevention. Results showed that 19% of participants reported reading or talking to someone about cervical cancer and 23% about Pap smear tests. Similarly, a study conducted in Syria among women assessing the level of knowledge of cervical cancer, HPV infection, and vaccines found that less than a third of participants had heard of HPV infection and vaccines against cervical cancer [19]. The current study also assesses why participants would not obtain a Pap-smear test. Responses included fear (82%), embarrassment (78%), too costly (77%), "don't believe in Pap smear test" (59%), "don't have time" (44%), "might be painful" (43%), and lack of women doctor (26%). These reasons are not uncommon among women in underdeveloped and developed countries. Similarly, a study conducted in South Korea, among college women with moderate knowledge of cervical cancer, but low levels of knowledge regarding cervical cancer screening found, that cultural barriers rank high in barriers to screening (social stigma), second, lack of knowledge, third, psychosocial barriers such as discomfort, lack of women doctors, and last, practical barriers such as time consuming or lack of time. [20]. Although cultural barriers were not measured in this study, based on the participant demographics, cultural barriers would be present in our study group and perhaps rank high based on results from previously reported studies done among Pakistani women [21]. One study, in particular, was performed in Norway and reported that Pakistani women living in Norway, which has one of the lowest cervical cancer mortalities in the world, reported similar barriers to screening among Pakistani women, mainly stigma and misconceptions surrounding the disease and lack of trust of healthcare providers [22] The encompassing goal of the study was to encourage women participants to plan to obtain a Pap smear test. Prior to the presentation, 54% of participants responded that they do not plan to have a Pap smear test within the next 6 months", and 44% said that they "don't know". After the cervical cancer awareness presentation, the level of knowledge regarding cervical cancer signs and symptoms, HPV vaccination, and early cancer detection and treatment outcome were significantly higher. The average knowledge percentage score was 8% pre-presentation compared to 53% post-presentation (p < 0.01), a 45% increase from baseline. The improvement in knowledge and awareness is consistent with similar cervical cancer interventional studies [23]. After the presentation, 31% of participants said they plan to have a Pap-smear test in the next 6 months (p<0.01). Other studies reported a similar increase in positive attitudes toward Pap smear testing [24-25]. However, despite the increase in cervical cancer knowledge and awareness, 38% of women said they "do not plan" to have a Pap smear test, and 30% "don't know". Part of the hesitancy or uncertainty can be cultural, lack of health insurance, education level, and beliefs about cervical cancer.

During the enrollment and interview process, many women expressed frustration with the lack of access to healthcare resources and facilities. Some women shared that they were scared of being diagnosed with cervical cancer. Some women expressed shyness regarding obtaining a Pap smear test, and few shared their belief's "myths" regarding cervical cancer. We did not assess cultural barriers and social determinants of health such as transportation and health care access and resources. For a large percentage of women in the study, this was the first time they heard about cervical cancer. Our study seems to support previous studies, and our findings are similar; thus, additional educational and awareness efforts are important because these types of outreach programs help to inform and educate women about the risks of cervical cancer while encouraging women to obtain a Pap-smear test and HPV vaccination.

## Conclusions

Cervical cancer is on the rise globally. All women are at risk for cervical cancer; however, low-income and low-educated women, particularly in developing countries, are at higher risk for cervical cancer infections and complications. Awareness about cervical cancer risk, detection, and prevention (HPV vaccines) are important to save lives. Our study suggests that pharmacy students' health outreach presentation about cervical cancer is an effective educational tool and that overall improvement in knowledge and awareness about cervical cancer encourages women to obtain Pap-smear tests among Pakistani women.
